# Relationship of TMAO levels with vascular function in patients with hypertension

**DOI:** 10.1038/s41440-025-02346-1

**Published:** 2025-09-24

**Authors:** Takayuki Yamaji, Yuji Takaeko, Farina Mohamad Yusoff, Shinji Kishimoto, Masato Kajikawa, Yukiko Nakano, Takanori Harada, Takahiro Harada, Aya Mizobuchi, Yusuke Saito, Shunsuke Tanigawa, Tatsuya Maruhashi, Ayumu Nakashima, Yukihito Higashi

**Affiliations:** 1 Center for Radiation Disaster Medical Science, Research Institute for Radiation Biology and Medicine, Hiroshima University, Hiroshima, Japan; 2 Department of Cardiovascular Regeneration and Medicine, Research Institute for Radiation Biology and Medicine, Hiroshima University, Hiroshima, Japan; 3 Department of Cardiology, Miyoshi Central Hospital, Hiroshima, Japan; 4 Division of Regeneration and Medicine, Medical Center for Translational and Clinical Research, Hiroshima University Hospital, Hiroshima, Japan; 5 Department of Cardiovascular Medicine, Hiroshima University Graduate School of Biomedical Sciences, Hiroshima, Japan; 6 Natural Science Center for Basic Research and Development, Hiroshima University, Higashi Hiroshima, Japan; 7 Department of Nephrology, Graduate School of Medicine, University of Yamanashi, Yamanashi, Japan

**Keywords:** Trimethylamine-N-oxide, Endothelial function, Flow-mediated vasodilation, Nitroglycerine-induced vasodilation, Morning hypertension

## Abstract

Increased trimethylamine-N-oxide (TMAO) level is a known risk factor for hypertension. Hypertension is associated with vascular dysfunction. Recently, it has been shown that endothelial function is impaired in relation to an increase in TMAO level. However, there is no information on the relationship between TMAO levels and vascular function in patients with hypertension. The purpose of this study was to evaluate the independent variables for circulating TMAO levels and to evaluate the relationships of circulating TMAO levels with vascular function assessed by flow-mediated vasodilation (FMD) and nitroglycerine-induced vasodilation (NID) in patients with hypertension. This study was a cross-sectional study. A total of 333 subjects with hypertension were enrolled in this study. Log TMAO levels were significantly correlated with age (*r* = 0.24, *p* < 0.01), low-density lipoprotein cholesterol (r = −0.15, *p* < 0.01), creatinine (*r* = 0.28, *p* < 0.01), estimated glomerular filtration rate (eGFR) (*r* = −0.28,* p* < 0.01), and uric acid (*r* = 0.13, *p* = 0.02). Multiple linear regression analysis revealed that age (*β* = 0.17, *p* = 0.03), eGFR (*β* = −0.22, *p *< 0.01) and receiving three or more kinds of antihypertensive drugs (*β* = 0.15, *p* = 0.02) were independent predictors of log TMAO levels. FMD (*r* = −0.11, *p* = 0.04) and NID (*r* = −0.11, *p* = 0.04) were significantly correlated with log TMAO levels. After adjustment for confounding factors for vascular function, log TMAO level was not an independent predictor of FMD and NID. These findings suggest that circulating TMAO levels are associated with age, eGFR, and receiving three or more kinds of antihypertensive drugs but not with vascular function in patients with hypertension. Clinical Trial Registry Information**:** http://www.umin.ac.jp (UMIN000003409).

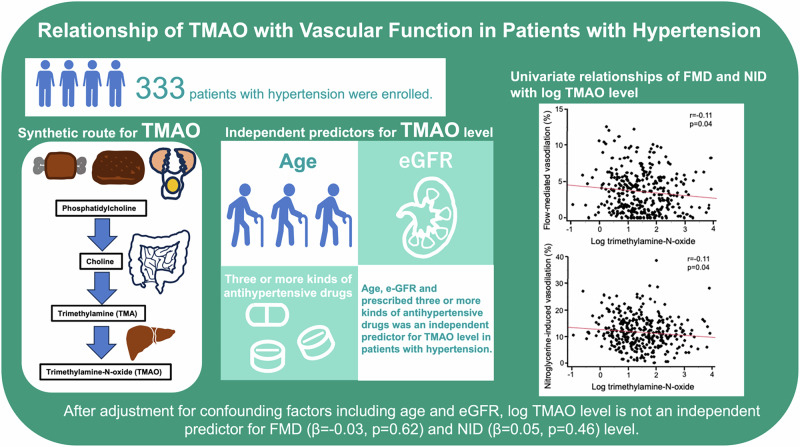

## Introduction

The gut microbiota consists of non-pathogenic bacteria that are involved in the digestion and absorption of food and produces several kinds of metabolites. Phosphatidylcholine, which is contained in red meat, eggs and liver, is hydrolyzed in the intestine to choline. Choline is converted into trimethylamine (TMA) by the gut microbiota. TMA is transported to the liver through the portal vein and metabolized to trimethylamine N-oxide (TMAO). TMAO circulates in the blood and promotes atherosclerosis [1]. A previous study showed that dietary supplementation with TMAO increased systolic blood pressure through an increase in advanced glycation end-products in mice [2]. A Mendelian randomization trial also showed a higher TMAO level was associated with higher systolic blood pressure [3]. There appears to be a potential association between TMAO and hypertension.

Endothelial dysfunction is the initial step in the progression of atherosclerosis, finally leading to cardiovascular events. The most common noninvasive method for assessing endothelial function is measurement of flow-mediated vasodilation (FMD) in the brachial artery. FMD is decreased by oxidative stress through reducing nitric oxide bioavailability, which is caused by traditional cardiovascular risks such as hypertension, dyslipidemia, diabetes mellitus and smoking [4–7]. Our previous study showed that regardless of systolic blood pressure levels, FMD was significantly lower in patients with hypertension who were receiving three or more kinds of antihypertensive drugs than in patients with hypertension who were receiving one kind or two kinds of antihypertensive drugs [8]. It is well known that FMD is an independent predictor of cardiovascular events [9]. Measurement of FMD is a useful method for assessing current cardiovascular event risks. Measurement of nitroglycerine-induced vasodilation (NID) has been used as a control test for FMD. Vascular smooth muscle function assessed by NID is impaired in relation to the state of progression of atherosclerosis. The most common noninvasive method for assessing vascular smooth muscle function is measurement of NID in the brachial artery [10]. NID is also known to be a useful marker of cardiovascular event risks. In addition, a combination of FMD and NID is more strongly associated with cardiovascular events than is FMD alone [11].

Previous studies showed that a higher TMAO level was associated with higher systolic blood pressure [2, 3]. However, there was no information on the effects of antihypertensive drug treatment on TMAO levels in patients with hypertension. Previous studies also showed that several medication therapies altered the human gut microbiota [12, 13], but it has remained uncertain whether antihypertensive drugs alter TMAO levels. A few studies have shown the relationship between TMAO and vascular function in patients with angina pectoris and patients with periodontitis [14, 15]. However, there is still no information on the relationship between TMAO level and vascular function assessed by FMD and NID in patients with hypertension. Therefore, in the present study, we evaluated the relationships of circulating TMAO level with vascular function assessed by FMD and NID in patients with hypertension.

## Methods

### Study subjects

A total of 333 subjects with hypertension who underwent blood tests and underwent a health checkup at Hiroshima University between January 2017 and February 2020 were enrolled in this study. Hypertension was defined as the use of antihypertensive drugs or systolic blood pressure of more than 140 mmHg or diastolic blood pressure of more than 90 mmHg measured in a sitting position on at least three occasions. Dyslipidemia was defined according to the third report of the National Cholesterol Education Program [16]. Diabetes mellitus was defined according to the American Diabetes Association recommendation [17]. Cardiovascular disease (CVD) was defined as coronary heart disease and cerebrovascular disease. Coronary heart disease included angina pectoris, prior myocardial infarction, and unstable angina. Cerebrovascular disease included ischemic stroke, hemorrhagic stroke, and transient ischemic attack. The Ethics Committee of Hiroshima University approved the study protocol. Written informed consent for participation in this study was obtained from all participants. The protocol was registered in the University Hospital Medical Information Network Clinical Trials Registry (UMIN000003409).

### Study protocol

This study was a cross-sectional study. To evaluate the association between the number of antihypertensive drugs being received and circulating TMAO levels, the subjects were divided into four groups based on information on the number of antihypertensive drugs received: no antihypertensive drugs received, one kind of antihypertensive drug, two kinds of antihypertensive drugs and three of more kinds of antihypertensive drugs. The subjects were also divided into two groups: one group of subjects who received less than three kinds of antihypertensive drugs and one group of subjects who received three or more kinds of antihypertensive drugs. We assessed the relationships between the number of antihypertensive drugs received and log TMAO level using propensity score matching. Next, we assessed the relationships of TMAO level with vascular function. The subjects fasted overnight and abstained from drinking alcohol, smoking, and taking caffeine and antioxidant vitamins for at least 12 h before the study. The participants were kept in the supine position in a quiet, dark, air-conditioned room (constant temperature of 22 °C to 25 °C) throughout the study. A 23-gauge polyethylene catheter was inserted into the deep antecubital vein to obtain blood samples. After maintaining the supine position for 30 minutes, FMD and NID were measured. The observers were blind to the form of examination. We extracted serum from a blood sample, and it was stored at −80 °C until later analysis of TMAO. Measurement of plasma TMAO levels was conducted by using stable isotope dilution liquid chromatography coupled with online tandem mass spectrometry (LC/MS/MS) [18].

### Measurements of FMD and NID

A high-resolution linear artery transducer was coupled to computer-assisted analysis software (UNEXEF18G, UNEX Co., Nagoya, Japan) that used an automated edge detection system for measurement of the brachial artery diameter [10]. A blood pressure cuff was placed around the forearm of each subject. The brachial artery was scanned longitudinally 5 to 10 cm above the elbow. When the clearest B-mode image of the anterior and posterior intimal interfaces between the lumen and vessel wall was obtained, the transducer was held at the same point throughout the scan by using a special probe holder (UNEX Co.) to ensure consistency of the imaging. Depth and gain settings were set to optimize the images of the arterial lumen wall interface. When the tracking gate was placed on the intima, the artery diameter was automatically tracked, and the waveform of diameter changes over the cardiac cycle was displayed in real time using the FMD mode of the tracking system. This allowed the ultrasound images to be optimized at the start of the scan and the transducer position to be adjusted immediately for optimal tracking performance throughout the scan. Pulsed Doppler flow was assessed at baseline and during peak hyperemic flow, which was confirmed to occur within 15 s after cuff deflation. Blood flow velocity was calculated from the color Doppler data and was displayed as a waveform in real time. Baseline longitudinal images of the artery were acquired for 30 s, and then the blood pressure cuff was inflated to 50 mm Hg above systolic pressure for 5 min. The longitudinal image of the artery was recorded continuously until 5 min after cuff deflation. Pulsed Doppler velocity signals were obtained for 20 s at baseline and for 10 s immediately after cuff deflation. Changes in brachial artery diameter were immediately expressed as percentage change relative to the vessel diameter before cuff inflation. FMD was automatically calculated as the percentage change in peak vessel diameter from the baseline value. Percentage of FMD [(Peak diameter–Baseline diameter)/Baseline diameter] was used for analysis. Blood flow volume was calculated by multiplying the Doppler flow velocity (corrected for the angle) by heart rate and vessel cross-sectional area (−*r*^2^). Reactive hyperemia was calculated as the maximum percentage increase in flow after cuff deflation compared with baseline flow.

The response to nitroglycerine was used for assessment of endothelium-independent vasodilation [10]. After acquiring baseline rest images for 30 s, a sublingual tablet (nitroglycerine, 75 µg) was given, and imaging of the artery was done continuously for five minutes. NID was automatically calculated as a percentage change in peak vessel diameter from the baseline. Percentage of NID [(Peak diameter–Baseline diameter)/Baseline diameter] was used for analysis. Inter- and intra-coefficients of variation for the brachial artery diameter were 1.6% and 1.4%, respectively, in our laboratory.

### Statistical analysis

Results are presented as means ± SD or medians (interquartile range). All reported p values were 2-sided, and a p value of <0.05 was considered statistically significant. Categorical values were compared by means of the chi-square test. Continuous variables were compared among multiple groups by using analysis of variance (ANOVA). Comparisons between the groups categorized according to TMAO levels were carried out using ANOVA with Tukey’s post hoc test. Univariate linear regression analyses were performed to assess the relationships among the variables. Multivariable linear regression analysis was performed to identify independent variables associated with log TMAO levels, FMD, and NID. In a previous study, we showed that age, sex, body mass index (BMI), presence of hypertension, dyslipidemia, or diabetes mellitus, and current smoking were independent predictors for FMD [4]. Therefore, in the present study, we adjusted these established confounding factors for vascular function and estimated glomerular filtration rate (eGFR). Age, sex, BMI, heart rate, systolic blood pressure, triglycerides, high-density lipoprotein cholesterol (HDL-C), total cholesterol, eGFR, hemoglobin A1c, prescribed three kinds of antihypertensive drugs, medication with lipid-lowering drugs, medication with anti-diabetic drugs, presence of CVD and current smoking were entered into the multivariable linear regression analysis for log TMAO levels. Age, sex, BMI, heart rate, systolic blood pressure, triglycerides, HDL-C, total cholesterol, eGFR, hemoglobin A1c, prescribed three kinds of antihypertensive drugs, medication with lipid-lowering drugs, medication with anti-diabetic drugs, presence of CVD, current smoking and log TMAO levels were entered into the multivariable linear regression analysis for FMD and NID. As a sensitivity analysis, propensity score analysis was used to minimize the selection bias for evaluation of the relationship between prescribed number of antihypertensive drugs and vascular function. The propensity score was calculated for each patient on the basis of logistic regression analysis of the probability within groups stratified by the number of prescribed antihypertensive drugs using clinical variables including age, sex, BMI, systolic blood pressure, diastolic blood pressure, heart rate, total cholesterol, triglycerides, eGFR, blood glucose, current smoking (yes or no), medication with lipid-lowering drugs (yes or no), medication with antidiabetic drugs and presence of CVD (yes or no). All data were processed using JMP Pro. Ver 17.0 software (SAS Institute, Cary, NC, USA).

## Results

### Baseline characteristics of the subjects

The baseline characteristics of the 333 patients are summarized in Table S1. The mean age of the subjects was 60 ± 14 years. The 333 subjects included 196 men (58.9%). Among the subjects, 230 (69.1%) had dyslipidemia, 75 (22.5%) had diabetes mellitus, 49 (14.7%) had previous CVD and 66 (19.9%) were current smokers. The mean FMD value was 3.6 ± 2.8% and the mean NID value was 11.7 ± 5.9%. The mean Log TMAO level was 1.5 ± 0.9.

### Univariate relationships between log TMAO level and absolute TMAO level and variables

Table 1 shows the univariate relationships between log TMAO level and variables. Log TMAO level was significantly correlated with age (*r* = 0.24, *p* < 0.01), systolic blood pressure (*r* = −0.12, *p* = 0.03), diastolic blood pressure (*r* = −0.13, *p* = 0.02), low-density lipoprotein cholesterol (LDL-C) (*r* = −0.15, *p* < 0.01), creatinine (*r* = 0.28, *p *< 0.01), eGFR (*r* = −0.28, *p* < 0.01), uric acid (*r* = 0.13, *p* = 0.02) and number of prescribed antihypertensive drugs (*r* = 0.20, *p* < 0.01). Absolute TMAO level was significantly correlated with age (r = 0.19, *p* < 0.01), diastolic blood pressure (*r* = −0.12, *p* = 0.03), LDL-C (*r* = −0.14, *p* = 0.01), creatinine (*r* = 0.35, *p* < 0.01), eGFR (*r *= −0.27, *p* < 0.01), and number of prescribed antihypertensive drugs (*r* = 0.16, *p* < 0.01).

**Table 1:** Univariate analysis of relationships among log TMAO level and variables

Variables	Log TMAO		TMAO	
	*r*	*P* value	*r*	*P* value
Age, year	0.24	<0.01	0.19	<0.01
Body mass index, kg/m^2^	−0.24	0.67	−0.01	0.91
Heart rate, bpm	−0.10	0.06	−0.08	0.14
Systolic blood pressure, mmHg	−0.12	0.03	−0.10	0.08
Diastolic blood pressure, mmHg	−0.13	0.02	−0.12	0.03
Total cholesterol, mg/dL	−0.06	0.35	−0.07	0.28
Triglycerides, mg/dL	<−0.01	0.88	0.001	0.99
HDL-C, mg/dL	−0.04	0.51	−0.05	0.34
LDL-C, mg/dL	−0.15	<0.01	−0.14	0.01
Creatinine, mg/dL	0.28	<0.01	0.35	<0.01
eGFR, mL/min/1.73 m^2^	−0.28	<0.01	−0.27	<0.01
Uric acid, mg/dL	0.13	0.02	0.07	0.19
Glucose, mg/dL	<0.01	0.98	0.02	0.71
Hemoglobin A1c, %	0.04	0.52	−0.005	0.93
Number of prescribed antihypertensive drugs	0.20	<0.01	0.16	<0.01

Univariate analysis of the relations between FMD, NID, log TMAO level and variables (Pearson’s correlation analysis)

*TMAO* indicates trimethylamine-N-oxide, *HDL-C* high-density lipoprotein cholesterol, *LDL-C* low-density lipoprotein cholesterol, *eGFR* estimated glomerular filtration rate

### Relationships between the number of prescribed antihypertensive drugs and TMAO levels

**Fig. 1:**
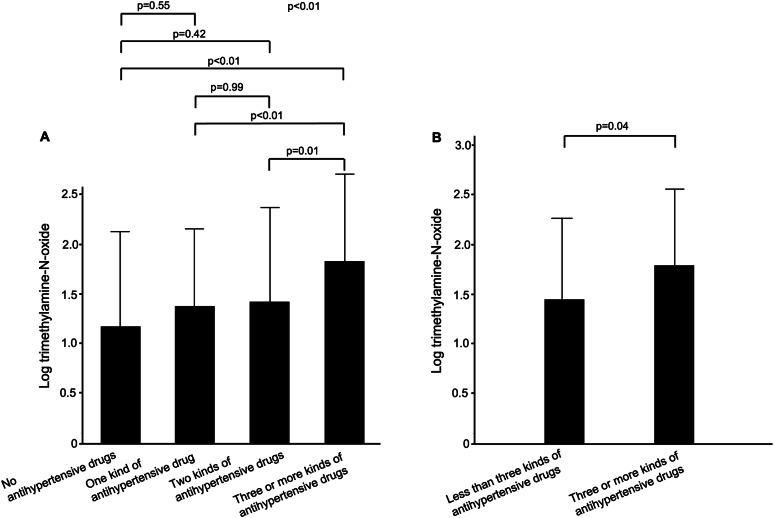
Bar graphs show log trimethylamine-N-oxide levels in four groups according to the number of prescribed antihypertensive drugs (**A**) and log trimethylamine-N-oxide levels in patients with who were receiving less than three kinds of antihypertensive drugs and patients who were prescribed three or more kinds of antihypertensive drugs in a propensity score-matched population (**B**)

Next, we divided the subjects into four groups according to their prescribed antihypertensive drugs: no prescribed antihypertensive drug group, one kind of antihypertensive drug group, two kinds of antihypertensive drugs group, and three or more kinds of antihypertensive drugs group. The baseline characteristics are summarized in Table 2. There were significant differences between the four groups in age, sex, heart rate, systolic blood pressure, diastolic blood pressure, total cholesterol, creatinine, LDL-C, creatinine, eGFR, uric acid, presence of diabetes mellitus, use of antihypertensive drugs, use of lipid-lowering drugs, use of anti-diabetic drugs, FMD levels, NID level, and log TMAO level. There was no significant difference in systolic blood pressure between the three or more kinds of antihypertensive drugs group and the two kinds of antihypertensive drugs group (*p* = 0.29). Systolic blood pressure was significantly higher in the no prescribed antihypertensive drug group than in the one kind of antihypertensive drug group (*p* < 0.01), two kinds of antihypertensive drugs group (*p* < 0.01) and three kinds of antihypertensive drugs group (*p* < 0.01). Log TMAO level was significantly higher in the three or more kinds of antihypertensive drugs group than in the no prescribed antihypertensive drug group (*p* < 0.01), one kind of antihypertensive drug group (*p* < 0.01) and two kinds of antihypertensive drugs group (*p* = 0.01) (Fig. 1A).

**Table 2:** Clinical characteristics of subjects

Variables	No antihypertensive drug (*n* = 33)	One kind of antihypertensive drug (*n* = 124)	Two kinds of antihypertensive drugs (*n* = 110)	Three or more kinds of antihypertensive drugs (*n* = 66)	*P* value
Age, yr	54 ± 13	58 ± 14	61 ± 14	64 ± 13	<0.01
Men, *n* (%)	19 (57.6)	60 (48.4)	68 (61.8)	49 (74.2)	<0.01
Body mass index, kg/m^2^	24.7 ± 3.9	24.7 ± 4.3	25.9 ± 4.0	25.8 ± 4.2	0.09
Heart rate, bpm	73 ± 13	74 ± 13	70 ± 10	68 ± 12	<0.01
Systolic blood pressure, mmHg	148 ± 13	131 ± 16	127 ± 17	132 ± 19	<0.01
Diastolic blood pressure, mmHg	92 ± 11	82 ± 11	77 ± 12	80 ± 12	<0.01
Total cholesterol, mg/dL	207 ± 34	199 ± 35	189 ± 32	186 ± 40	0.02
Triglycerides, mg/dL	124 (101, 234)	115 (82, 191)	124 (85, 177)	142 (94, 208)	0.57
HDL-C, mg/dL	59 ± 16	59 ± 18	59 ± 15	56 ± 15	0.48
LDL-C, mg/dL	123 ± 27	115 ± 29	108 ± 30	107 ± 27	0.02
Creatinine, mg/dL	0.75 ± 0.15	0.76 ± 0.17	0.81 ± 0.17	0.99 ± 0.61	<0.01
eGFR, mL/min/1.73 m^2^	78 ± 15	73 ± 16	70 ± 15	66 ± 22	<0.01
Uric acid, mg/dL	5.9 ± 1.4	5.5 ± 1.4	6.0 ± 1.3	6.1 ± 1.2	<0.01
Glucose, mg/dL	114 ± 26	107 ± 22	106 ± 22	114 ± 26	0.08
Hemoglobin A1c, %	5.3 ± 0.6	5.5 ± 0.9	5.5 ± 0.8	5.5 ± 0.7	0.69
Medical history, *n* (%)					
Dyslipidemia	17 (51.5)	84 (67.7)	82 (74.6)	47 (71.2)	0.09
Diabetes mellitus	3 (9.1)	24 (19.4)	26 (23.6)	22 (33.3)	0.03
CVD	2 (6.1)	17 (13.7)	15 (13.6)	15 (22.7)	0.14
Current smoker, *n* (%)	12 (36.4)	19 (15.3)	22 (20.0)	13 (20.0)	0.06
Medication, *n* (%)					
Antihypertensive drugs	0 (0)	124 (100)	110 (100)	66 (100)	NA
Calcium channel blockers	0 (0)	80 (64.5)	106 (96.4)	62 (93.9)	<0.01
ARBs/ACEIs	0 (0)	21 (16.9)	70 (63.6)	59 (89.4)	<0.01
α-Blockers	0 (0)	1 (0.8)	9 (8.2)	11 (16.7)	<0.01
β-Blockers	0 (0)	0 (0)	5 (4.6)	17 (25.8)	<0.01
Diuretics	0 (0)	1 (0.8)	3 (2.7)	37 (56.1)	<0.01
Aldosterone antagonists	0 (0)	21 (16.9)	27 (24.6)	23 (34.9)	<0.01
Lipid-lowering drugs	4 (12.1)	49 (39.5)	47 (42.7)	26 (39.4)	0.01
Anti-diabetic drugs	1 (3.0)	19 (15.3)	14 (12.7)	16 (24.2)	0.04
FMD, %	5.0 ± 3.0	3.9 ± 2.7	3.1 ± 2.8	3.1 ± 2.4	<0.01
NID, %	13.1 ± 4.6	12.9 ± 6.5	10.8 ± 5.7	10.3 ± 4.8	<0.01
Log TMAO	1.2 ± 0.9	1.4 ± 0.8	1.4 ± 0.9	1.8 ± 0.9	<0.01
TMAO, μM	2.5 (1.6, 6.1)	3.7 (2.3, 6.7)	4.0 (2.1, 8.2)	5.8 (3.3, 10.7)	<0.01

The results are presented as means and ±SD or medians with interquartile ranges

*HDL-C* high-density lipoprotein cholesterol, *LDL-C* low-density lipoprotein cholesterol, *eGFR* estimated glomerular filtration rate, *CVD* cardiovascular disease, *ARBs* angiotensin II receptor blockers, *ACEIs* angiotensin-converting enzyme inhibitors, *FMD* flow-mediated vasodilation, *NID* nitroglycerine-induced vasodilation, *TMAO* trimethylamine N-oxide

Table 3 shows multivariable linear relationships between log TMAO level and variables. Multiple linear regression analysis revealed that age (*β* = 0.17, *p* = 0.03), eGFR (*β* = −0.22, *p* < 0.01) and prescribed three or more kinds of antihypertensive drugs (*β* = 0.15, *p* = 0.02) were independent predictors of log TMAO level. Finally, propensity score matching analysis was performed to create matched pairs between the less than three kinds of antihypertensive drug group and the three or more kinds of antihypertensive drugs group. The baseline characteristics of the matched pairs are summarized in Table S2. Log TMAO level was significantly higher in the three or more kinds of antihypertensive drugs group than in the less than three kinds of antihypertensive drugs group (1.4 ± 0.8 versus 1.8 ± 0.8; *p* = 0.04) (Fig. 1B).

**Table 3:** Multivariate analysis of relationships between log TMAO level and variables

Variables	Log TMAO			
	*β*	VIF	*t* value	*P* value
Age, year	0.17	1.79	2.13	0.03
Body mass index, kg/m^2^	−0.02	1.37	−0.31	0.76
Heart rate, bpm	0.04	1.20	0.53	0.60
Systolic blood pressure, mmHg	−0.09	1.29	−1.37	0.17
Triglycerides, mg/dL	0.06	1.40	0.77	0.44
HDL-C, mg/dL	−0.02	1.66	−0.27	0.78
Total cholesterol, mg/dL	−0.05	1.45	−0.70	0.49
eGFR, mL/min/1.73 m^2^	−0.22	1.36	−3.19	<0.01
Hemoglobin A1c, %	0.02	1.57	0.27	0.79
Men	0.09	1.26	1.30	0.20
Three or more kinds of antihypertensive drugs	0.15	1.15	2.31	0.02
Lipid-lowering drug treatment	0.02	1.57	0.32	0.75
Anti-diabetic drug treatment	0.03	1.64	0.42	0.68
Past CVD	−0.03	1.44	−0.36	0.72
Current smoking	0.08	1.17	1.28	0.20

The adjusted *r*^2^ was 0.19

*TMAO* trimethylamine-N-oxide, *HDL-C* high-density lipoprotein cholesterol, *eGFR* estimated glomerular filtration rate, *CVD* cardiovascular disease

### Univariate analysis of relationships of FMD and NID with variables

**Fig. 2:**
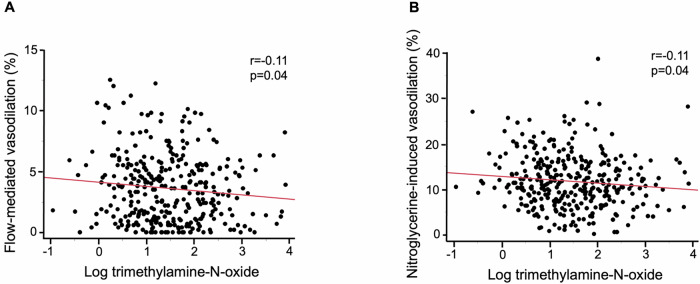
Scatter plots show relationships of log trimethylamine-N-oxide level with flow-mediated vasodilation (**A**) and nitroglycerine-induced vasodilation (**B**)

Table S3 shows univariate relationships of FMD and NID with variables. FMD was significantly correlated with age (*r* = −0.37, *p* < 0.01), systolic blood pressure (*r *= 0.21, *p* < 0.01), diastolic blood pressure (*r* = 0.32, *p* < 0.01), hemoglobin A1c (*r* = −0.16, *p* < 0.01), NID (*r* = 0.52, *p* < 0.01), and log TMAO level (*r* = −0.11, *p* = 0.04) (Fig. 2A). NID was significantly correlated with age (*r* = −0.36, *p* < 0.01), diastolic blood pressure (*r* = 0.27, *p* < 0.01), HDL-C (*r *= 0.16, *p *< 0.01), LDL-C (*r* = 0.14, *p* = 0.01), eGFR (*r* = 0.12, *p* = 0.04), uric acid (*r* = −0.15, *p* < 0.01), hemoglobin A1c (*r* = −0.13, *p* = 0.02), FMD (*r* = 0.52, *p* < 0.01), and log TMAO level (*r* = −0.11, *p* = 0.04) (Fig. 2B).

### Multivariate analysis of relationships among log TMAO levels, FMD, NID, and variables

Table 4 shows multivariable linear relationships among FMD, NID and variables. Multivariate linear regression analysis revealed that age (*β* = −0.22, *p* < 0.01) and systolic blood pressure (*β* = 0.19, *p* < 0.01) were independent predictors of FMD. Log TMAO level (*β* = −0.04, *p* = 0.53) was not an independent predictor of FMD. Multivariate linear regression analysis revealed that age (*β* = −0.28, *p* < 0.01), BMI (*β* = −0.16, *p* = 0.02), and past CVD (*β* = 0.17, *p* = 0.02) were independent predictors of NID. Log TMAO level (*β* < 0.01, *p* = 0.96) was not an independent predictor of NID.

**Table 4:** Multivariable analysis of relationships of FMD and NID with variables

Variables	FMD				NID			
	*β*	VIF	*t* value	*P* value	*β*	VIF	*t* value	*P* value
Age, year	−0.22	1.83	−2.68	<0.01	−0.28	1.82	−3.36	<0.01
Body mass index, kg/m^2^	−0.09	1.27	−1.30	0.20	−0.16	1.38	−2.27	0.02
Heart rate, bpm	0.07	1.21	1.00	0.32	0.04	1.21	0.61	0.54
Systolic blood pressure, mmHg	0.19	1.30	2.79	<0.01	0.03	1.31	0.42	0.67
Triglycerides, mg/dL	−0.03	1.40	−0.41	0.68	−0.04	1.38	−0.60	0.55
HDL-C, mg/dL	<0.01	1.66	0.00	1.00	0.08	1.66	1.08	0.28
Total cholesterol, mg/dL	−0.03	1.46	−0.03	0.70	0.03	1.47	0.41	0.69
eGFR, mL/min/1.73m^2^	−0.02	1.42	−0.25	0.81	<0.01	1.42	0.01	1.00
Hemoglobin A1c, %	−0.14	1.57	−1.90	0.06	−0.06	1.57	−0.75	0.45
Men	−0.09	1.27	−1.30	0.20	0.04	1.27	0.57	0.57
Three or more kinds of antihypertensive drugs	−0.01	1.18	−0.15	0.88	<0.01	1.18	0.04	0.96
Lipid-lowering drug treatment	0.08	1.47	1.13	0.26	−0.01	1.46	−0.09	0.93
Antidiabetic drug treatment	−0.06	1.64	−0.77	0.44	−0.06	1.64	−0.75	0.45
Past CVD	0.10	1.44	1.31	0.19	0.17	1.45	−2.38	0.02
Current smoking	−0.03	1.18	0.47	0.64	0.06	1.17	0.97	0.33
Log TMAO	−0.04	1.23	−0.63	0.53	<0.01	1.18	−0.04	0.96

The adjusted *r*^2^ was 0.21 in FMD and 0.18 in NID

*FMD* flow-mediated vasodilation, *NID* nitroglycerine-induced vasodilation, *HDL-C* high-density lipoprotein cholesterol, *eGFR* estimated glomerular filtration rate, *CVD* cardiovascular disease, *TMAO* trimethylamine-N-oxide

### Relationship between the presence of CVD and TMAO

Table S4 shows clinical characteristics of patients with and without CVD. There were significant differences in age, sex, heart rate, diastolic blood pressure, total cholesterol, LDL-C, creatinine, eGFR, hemoglobin A1c, presence of dyslipidemia, presence of diabetes mellitus, use of lipid-lowering drugs, use of anti-diabetic drugs, and NID between the two groups. FMD levels were similar in the CVD group and the non-CVD group (*p *= 0.12), whereas NID level were significantly lower in the CVD groups than in the non-CVD groups (*p* < 0.01). In the univariate analysis, the presence of CVD was not associated with log TMAO levels (*t* value = −1.72, *p* = 0.09).

### Relationship between blood pressure and TMAO

No significant correlation was observed between systolic blood pressure and log TMAO levels. Log TMAO level was significantly higher in the three or more kinds of antihypertensive drugs group than the other groups. Next, we assessed the relationships between the number of prescribed drugs and log TMAO levels. Log TMAO level was significantly higher in the three or more kinds of antihypertensive drugs group than in the other groups. Then, we divided subjects into two groups: low TMAO group and high TMAO group. Table S5 shows the baseline characteristics of the high TMAO group and the low TMAO group. There were significant differences in age, sex, heart, systolic blood pressure, diastolic blood pressure, LDL-C, creatinine, eGFR, uric acid, use of antihypertensive drugs, use of angiotensin receptor blockers/angiotensin-converting enzyme inhibitors, and number of hypertensive drugs between the two groups. Systolic blood pressure and diastolic blood pressure were significantly lower in the high TMAO group than in the low TMAO group, whereas the number of prescribed antihypertensive drugs was significantly higher in the high TMAO group than in the low TMAO group.

### Subgroup analysis based on age and sex

Finally, we assessed subgroup analysis stratified by age, sex, presence of dyslipidemia, presence of diabetes mellitus, current smoker, and prevalence of CVD. For FMD, interactions between log TMAO and sex, older age, presence of dyslipidemia, presence of diabetes mellitus, current smoker, and previous CVD were not statistically significant (*p* for interaction: 0.24, 0.95, 0.62, 0.57, 0.95, and 0.50, respectively) (Table S6). For NID, interactions between log TMAO and sex, older age, presence of dyslipidemia, presence of diabetes mellitus, current smoker, and previous CVD were not statistically significant (*p* for interaction: 0.64, 0.49, 0.69, 0.45, 0.39, and 0.77, respectively) (Table S7).

## Discussion

In the present study, we showed for the first time that circulating TMAO level was significantly higher in patients who were prescribed three or more kinds of antihypertensive drugs than in patients who were prescribed less than three kinds of antihypertensive drugs. Age, eGFR and prescribed three or more kinds of antihypertensive drugs were independent predictors of log TMAO level. Furthermore, we showed that log TMAO level significantly correlated with FMD and NID in patients with hypertension. However, after adjustment for confounding factors of vascular function, although age and eGFR were associated with TMAO level, there was no significant relationship of TMAO level with FMD or NID.

First, we assessed the associations between log TMAO level and variables. Univariate analysis showed that systolic blood pressure was negatively correlated with log TMAO level in patients with hypertension. This finding differs from previous studies showing that TMAO promotes arterial stiffness and elevates blood pressure [2]. Therefore, to evaluate the effects of antihypertensive drugs on TMAO levels, we assessed the number of prescribed antihypertensive drugs was significantly correlated with log TMAO level. There has been no study on the relationships of log TMAO level with number of prescribed antihypertensive drugs. Next, to evaluate the association between the number of prescribed antihypertensive drugs and TMAO levels, we divided the subjects into four groups and assessed log TMAO levels. Interestingly, log TMAO level was significantly higher in patients who were receiving three or more kinds of antihypertensive drugs than in the other groups. The possible reasons for the association between the number of prescribed antihypertensive drugs and log TMAO level are that TMAO increases vascular stiffness, leading to the need for a large number of antihypertensive drugs to achieve an appropriate blood pressure and that the effects of drugs on gut microbiota lead to an increase in TMAO level. Brunt et al. showed that TMAO increases oxidative stress and advanced glycation end products, leading to an increase in atrial wall stiffness and increase in blood pressure [2]. Previous studies showed that many pharmaceuticals influence the human gut microbiota [12, 13]. Previous studies also showed that TMAO levels were elevated in relation to impairment of renal function since TMAO is excreted by the kidneys [19, 20] and that TMAO levels were significantly correlated with age [20, 21]. Our study showed that age, eGFR and receiving three or more kinds of antihypertensive drugs were independent predictors of log TMAO level.

Finally, we investigated whether TMAO level was an independent predictor for FMD and NID. In the present study, TMAO levels were significantly correlated with FMD and NID. After adjustment for those established confounding factors and eGFR, log TMAO level was not an independent predictor for FMD and NID. In the present study, log TMAO levels were strongly correlated with age and eGFR. Therefore, when age and eGFR were included as adjustments for confounding factors in multivariate logistic regression analysis, TMAO level may have been a strong confounding factor of age and eGFR and thus was not selected as an independent predictor of FMD and NID. A few studies have shown the relationship between TMAO levels and FMD. In 122 patients with periodontitis, TMAO levels were higher and FMD levels were lower than those in patients without periodontitis [22]. Unfortunately, multivariate analysis to determine whether TMAO level is a determinant of vascular endothelial function has not been conducted. Although Chou et al. showed that TMAO level was an independent predictor for FMD in 81 patients with stable angina [14], their multivariable analysis did not include determinants of vascular endothelial function such as age and eGFR. However, they focused on patients with stable angina, and they enrolled patients who were older than the patients in our study. Therefore, the enrolled patients in their study had a narrow age range, and there was no significant correlation between age and FMD in univariate analysis.

This study has several limitations. First, this study was a cross-sectional study. The finding of a relationship between TMAO level and vascular function in the univariate analysis does not establish a causal relationship. In the multivariate analysis, we did not show an independent relationship between circulating TMAO level and vascular function. Second, unfortunately, we did not have information on choline and betaine. Information on choline and betaine levels would enable more specific conclusions concerning the role of TMAO in vascular function to be drawn. Third, this study was conducted in Japanese. Previous reports showed that gut bacteria levels vary by race and dietary habits, and, even in the same race, vary by age and BMI [23, 24]. Therefore, our results might not be applicable to other races. Further studies are needed to establish the association between TMAO and vascular function in a general population, including various races. Fourth, we cannot completely exclude the possibility of effects of confounding factors for vascular function, such as dietary and exercise habits, on TMAO and vascular function. Fifth, although TMAO level were higher in the CVD group than non-CVD group, no statistically significant association was observed between the presence of CVD and TMAO levels. In the present study, the number of patients with CVD was small. Therefore, we cannot deny the possibility that the small sample size may have influenced the statistical analyses. Sixth, the association between higher blood pressure and lower TMAO levels seems counterintuitive. Therefore, we assessed the relationships between systolic blood pressure and log TMAO levels in patients with hypertension who are not taking antihypertensive drug treatment. Unfortunately, the number of patients with hypertension who are not taking antihypertensive drug treatment was small. No significant correlation was observed between systolic blood pressure and log TMAO levels. Moreover, we assessed the relationships between the number of prescribed drugs and log TMAO levels. Log TMAO level was significantly higher in the three or more kinds of antihypertensive drugs group than in the other groups. In addition, we divided subjects into two groups: low TMAO group and high TMAO group. Systolic blood pressure and diastolic blood pressure were significantly lower in the high TMAO group than in the low TMAO group, whereas the number of prescribed antihypertensive drugs was significantly higher in the high TMAO group than in the low TMAO group. These findings suggest the hypothesis that elevated TMAO is associated with greater antihypertensive drug usage. Patients with hypertension who require a combination of three or more antihypertensive drugs to achieve blood pressure control may have experienced greater cumulative exposure to blood pressure compared to those who treated with one or two antihypertensive drugs, which may contribute to the higher TMAO levels. Further study is needed to determine whether the relationship between endothelial function and blood pressure levels differs across classes of antihypertensive medications. Seventh, endothelial dysfunction assessed by FMD reflects the initial step for atherosclerosis and vascular smooth muscle dysfunction assessed by NID reflects an advanced stage of atherosclerosis. We previously showed that both FMD and NID decreased in relation to cumulative cardiovascular risk factors and significantly correlated with cardiovascular risk factors [10]. Although FMD tended to be higher in the CVD group compared to the non-CVD group, the difference was not statistically significant. In contrast, NID was significantly higher in the CVD group than in the non-CVD group. In the present study, we focused on patients with hypertension. The precise reason why FMD does not appear to be attenuated in hypertensive patients with CVD, compared to those without CVD, remains uncertain. Most patients with hypertension were receiving antihypertensive medications. Previously, we reported that in hypertensive patients undergoing antihypertensive treatment, endothelial function may be masked by the pharmacological effects of these medications, making it difficult to accurately assess vascular function [8].

In conclusion, age, eGFR and receiving three or more kinds of antihypertensive drugs are independent predictors of circulating TMAO level in patients with hypertension. TMAO level significantly correlated with FMD and NID in patients with hypertension. However, after adjustment for confounding factors for vascular function, TMAO level was not an independent predictor of FMD and NID.

## Supplementary information

Supplemental Data
