# Supplementary material for: Relationship of TMAO levels with vascular function in patients with hypertension
Source: Hypertens Res. 2025 Sep 24;48(12):3198–208. doi: 10.1038/s41440-025-02346-1 (PMC12678174; doi:10.1038/s41440-025-02346-1)
Supplement: Supplementary file 1 — Supplemental Data [file 41440_2025_2346_MOESM1_ESM.docx]

**Supplemental Data**

**Relationship** **of TMAO Levels with Vascular Function in Patients with Hypertension**

Takayuki Yamaji, MD, PhD;^1,2^ Yuji Takaeko, MD, PhD;^3^ Farina Mohamad Yusoff, MBBS, PhD, FSVM; ^2^Shinji Kishimoto, MD, PhD;^2^ Masato Kajikawa, MD, PhD;^4^Yukiko Nakano, MD, PhD;^5^ Takanori Harada, PhD;^6^ Takahiro Harada, MD, PhD; ^2^ Aya Mizobuchi, PhD;^2^ Yusuke Saito, MS;^2^ Shunsuke Tanigawa, MS;^2^ Tatsuya Maruhashi, MD, PhD; ^2^ Ayumu Nakashima, MD, PhD;^7^ Yukihito Higashi, MD, PhD, FAHA^1,2,4^

1 Center for Radiation Disaster Medical Science, Research Institute for Radiation Biology and Medicine, Hiroshima University, Hiroshima, Japan

2 Department of Cardiovascular Regeneration and Medicine, Research Institute for Radiation Biology and Medicine, Hiroshima University, Hiroshima, Japan

3Department of Cardiology, Miyoshi Central Hospital, Hiroshima, Japan

4 Division of Regeneration and Medicine, Medical Center for Translational and Clinical Research, Hiroshima University Hospital, Hiroshima, Japan

5 Department of Cardiovascular Medicine, Hiroshima University Graduate School of Biomedical Sciences, Hiroshima, Japan

6 Natural Science Center for Basic Research and Development, Hiroshima University, Higashi Hiroshima, Japan

7 Department of Nephrology, Graduate School of Medicine, University of Yamanashi, Yamanashi, Japan.

Running title: TMAO and vascular function

Address for correspondence: Yukihito Higashi, MD, PhD, FAHA

Department of Cardiovascular Regeneration and Medicine,

Research Institute for Radiation Biology and Medicine, Hiroshima University

1-2-3 Kasumi, Minami-ku, Hiroshima 734-8551, Japan

Phone: +81-82-257-5831 Fax: +81-82-257-5831

E-mail: [yhigashi@hiroshima-u.ac.jp](mailto:yhigashi@hiroshima-u.ac.jp)

**Supplemental Table**

**Table S1.** Clinical Characteristics of Patients with Hypertension

| Variables | Total  (n=333) |
| --- | --- |
| Age, yr | 60±14 |
| Men, n (%) | 196 (58.9) |
| Body mass index, kg/m^2^ | 25.3±4.2 |
| Heart rate, bpm | 71±12 |
| Systolic blood pressure, mmHg | 132±18 |
| Diastolic blood pressure, mmHg | 81±12 |
| Total cholesterol, mg/dL | 194±36 |
| Triglycerides, mg/dL | 124 (87, 190) |
| HDL-C, mg/dL | 58±16 |
| LDL-C, mg/dL | 112±29 |
| Creatinine, mg/dL | 0.82±0.33 |
| eGFR, mL/min/1.73m^2^ | 71±17 |
| Uric acid, mg/dL | 5.8±1.4 |
| Glucose, mg/dL | 109±23 |
| Hemoglobin A1c, % | 5.5±0.8 |
| Medical history, n (%) |  |
| Dyslipidemia | 230 (69.1) |
| Diabetes mellitus | 75 (22.5) |
| CVD | 49 (14.7) |
| Current smoker, n (%) | 66 (19.9) |
| Medication, n (%) |  |
| Antihypertensive drugs | 300 (90.1) |
| Calcium channel blockers | 248 (74.4) |
| ARB/ACEIs | 150 (45.0) |
| α-Blockers | 21 (6.3) |
| β-Blockers | 22 (6.6) |
| Diuretics | 41 (12.3) |
| Aldosterone antagonists | 71 (21.3) |
| Lipid lowering drugs | 126 (37.8) |
| Anti-diabetic drugs | 50 (15.0) |
| FMD, % | 3.6±2.8 |
| NID, % | 11.7±5.9 |
| Log TMAO | 1.5±0.9 |
| TMAO, μM | 4.0 (2.3, 8.0) |

HDL-C indicates high-density lipoprotein cholesterol; LDL-C, low-density lipoprotein cholesterol; eGFR, estimated glomerular filtration rate; CVD, cardiovascular disease; ARB, angiotensin II receptor blockers; ACEIs, angiotensin-converting enzyme inhibitors; FMD, flow-mediated vasodilation; NID, nitroglycerine-induced vasodilation; TMAO, trimethylamine N-oxide. The results are presented as means and ± SD or medians with interquartile ranges.

**Table S2.** Clinical Characteristics of Patients with Hypertension Who Were Prescribed Less Than Three Kinds of Antihypertensive Drugs and Patients with Hypertension Who Were Prescribed Three or More Kinds of Antihypertensive Drugs in Matched Pairs

| Variables | Less than three kinds of antihypertensive drugs  (n=45) | Three or more kinds of antihypertensive drugs  (n=45) | P value |
| --- | --- | --- | --- |
| Age, yr | 65±13 | 66±12 | 0.69 |
| Men (%) | 38 (84.4) | 34 (75.6) | 0.29 |
| Body mass index, kg/m^2^ | 25.0±3.7 | 25.5±3.9 | 0.48 |
| Heart rate, bpm | 68±13 | 70±12 | 0.50 |
| Systolic blood pressure, mmHg | 127±21 | 130±20 | 0.56 |
| Diastolic blood pressure, mmHg | 78±15 | 78±11 | 0.78 |
| Total cholesterol, mg/dl | 185±35 | 185±42 | 0.94 |
| Triglycerides, mg/dl | 122 (87, 160) | 118 (94, 188) | 0.62 |
| HDL-C, mg/dL | 58±19 | 56±16 | 0.67 |
| LDL-C, mg/dL | 106±30 | 106±28 | 0.96 |
| Creatinine, mg/dL | 0.88±0.16 | 0.96±0.63 | 0.37 |
| eGFR, mL/min/1.73m^2^ | 67.5±13.7 | 66.0±19.0 | 0.67 |
| Uric acid, mg/dL | 6.0±1.4 | 5.9±1.0 | 0.83 |
| Glucose, mg/dL | 112±26 | 109±22 | 0.54 |
| Hemoglobin A1c, % | 5.8±1.1 | 5.5±0.7 | 0.22 |
| Medical history, n (%) |  |  |  |
| Dyslipidemia | 25 (55.6) | 32 (71.1) | 0.13 |
| Diabetes mellites | 16 (35.6) | 13 (28.9) | 0.50 |
| CVD, n (%) | 12 (26.7) | 10 (22.2) | 0.62 |
| Current Smoking, n (%) | 12 (26.7) | 7 (15.6) | 0.20 |
| Medication, n (%) |  |  |  |
| Calcium channel blockers | 33 (73.3) | 41 (91.1) | 0.03 |
| ARB/ACEIs | 20 (44.4) | 42 (93.3) | <0.001 |
| α-Blockers, | 1 (2.2) | 7 (15.6) | 0.03 |
| β-Blockers | 1 (2.2) | 10 (22.2) | <0.001 |
| Diuretics, | 1 (2.2) | 29 (64.4) | <0.001 |
| Aldosterone antagonists | 2 (4.4) | 11 (24.4) | <0.001 |
| Lipid lowering drugs | 19 (42.2) | 19 (42.2) | 1.00 |
| Antidiabetic drugs, n (%) | 12 (26.7) | 8 (17.8) | 0.31 |
| FMD, % | 2.8±2.2 | 2.7±2.3 | 0.81 |
| NID, % | 10.9±6.2 | 10.6±5.1 | 0.75 |
| Log TMAO | 1.4±0.8 | 1.8±0.8 | 0.04 |
| TMAO, μM | 4.1 (2.1, 8.2) | 5.6 (3.6, 9.4) | 0.05 |

HDL-C indicates high-density lipoprotein cholesterol; LDL-C, low-density lipoprotein cholesterol; eGFR, estimated glomerular filtration rate; CVD, cardiovascular disease; ARB, angiotensin II receptor blockers; ACEIs, angiotensin-converting enzyme inhibitors; FMD, flow-mediated vasodilation; NID, nitroglycerine-induced vasodilation; TMAO, trimethylamine N-oxide. The results are presented as means and ± SD or medians with interquartile ranges.

| Variables | FMD | | NID | |
| --- | --- | --- | --- | --- |
|  | r | P value | r | P value |
| Age, yr | -0.37 | <0.01 | -0.36 | <0.01 |
| Body mass index, kg/m^2^ | -0.01 | 0.83 | -0.05 | 0.39 |
| Heart rate, bpm | 0.04 | 0.47 | 0.08 | 0.14 |
| Systolic blood pressure, mmHg | 0.21 | <0.01 | 0.07 | 0.20 |
| Diastolic blood pressure, mmHg | 0.32 | <0.01 | 0.27 | <0.01 |
| Total cholesterol, mg/dL | 0.06 | 0.34 | 0.11 | 0.07 |
| Triglycerides, mg/dL | 0.03 | 0.60 | -0.01 | 0.88 |
| HDL-C, mg/dL | 0.05 | 0.36 | 0.16 | <0.01 |
| LDL-C, mg/dL | 0.10 | 0.07 | 0.14 | 0.01 |
| Creatinine, mg/dL | -0.04 | 0.41 | -0.04 | 0.50 |
| eGFR, mL/min/1.73m^2^ | 0.07 | 0.24 | 0.12 | 0.04 |
| Uric acid, mg/dL | -0.02 | 0.69 | -0.15 | <0.01 |
| Glucose, mg/dL | -0.07 | 0.23 | -0.08 | 0.13 |
| Hemoglobin A1c, % | -0.16 | <0.01 | -0.13 | 0.02 |
| FMD, % |  |  | 0.52 | <0.01 |
| NID, % | 0.52 | <0.01 |  |  |

**Table S3.** Univariate Analysis of Relationships of FMD and NID with Variables

HDL-C indicates high-density lipoprotein cholesterol; LDL-C, low-density lipoprotein cholesterol; eGFR, estimated glomerular filtration rate; FMD, flow-mediated vasodilation; NID, nitroglycerine-induced vasodilation.

Univariate analysis of the relations between FMD, NID, and variables (Pearson’s correlation analysis).

**Table S4.** Clinical Characteristics of Patients with and without CVD

| Variables | Non-CVD  (n=284) | CVD  (n=49) | P value |
| --- | --- | --- | --- |
| Age, yr | 58±14 | 68±9 | <0.01 |
| Men, n (%) | 152 (53.5) | 44 (89.8) | <0.01 |
| Body mass index, kg/m^2^ | 25.4±4.2 | 24.4±3.6 | 0.12 |
| Heart rate, bpm | 72±12 | 67±10 | <0.01 |
| Systolic blood pressure, mmHg | 132±18 | 130±15 | 0.44 |
| Diastolic blood pressure, mmHg | 81±12 | 77±12 | 0.03 |
| Total cholesterol, mg/dL | 197±35 | 180±35 | <0.01 |
| Triglycerides, mg/dL | 124 (86, 189) | 118 (93, 194) | 0.37 |
| HDL-C, mg/dL | 59±16 | 55±17 | 0.18 |
| LDL-C, mg/dL | 114±28 | 98±30 | <0.01 |
| Creatinine, mg/dL | 0.79±0.23 | 0.99±0.65 | <0.01 |
| eGFR, mL/min/1.73m^2^ | 72±17 | 66±18 | 0.01 |
| Uric acid, mg/dL | 5.8±1.4 | 5.9±1.2 | 0.61 |
| Glucose, mg/dL | 108±23 | 114±27 | 0.09 |
| Hemoglobin A1c, % | 5.4±0.7 | 5.7±1.2 | 0.04 |
| Medical history, n (%) |  |  |  |
| Dyslipidemia | 189 (66.6) | 41 (83.7) | 0.02 |
| Diabetes mellitus | 55 (19.4) | 20 (40.8) | <0.01 |
| Current smoker, n (%) | 58 (20.5) | 8 (16.3) | 0.50 |
| Medication, n (%) |  |  |  |
| Antihypertensive drugs | 253 (89.1) | 47 (95.9) | 0.14 |
| Calcium channel blockers | 207 (72.9) | 41 (83.7) | 0.11 |
| ARB/ACEIs | 123 (43.3) | 27 (55.1) | 0.13 |
| α-Blockers, | 20 (7.0) | 1 (2.0) | 0.18 |
| β-Blockers | 16 (5.6) | 6 (12.2) | 0.09 |
| Diuretics, | 31 (10.9) | 10 (20.4) | 0.06 |
| Aldosterone antagonists | 64 (22.5) | 7 (14.3) | 0.19 |
| Lipid lowering drugs | 87 (30.6) | 39 (79.6) | <0.01 |
| Anti-diabetic drugs | 37 (13.0) | 13 (26.5) | 0.01 |
| Number of hypertensive drugs | 1.6±1.0 | 1.9±0.9 | 0.09 |
| FMD, % | 4.7±2.8 | 3.0±2.4 | 0.12 |
| NID, % | 12.2±5.8 | 8.8±5.3 | <0.01 |
| Log TMAO | 1.4±0.9 | 1.7±0.8 | 0.09 |
| TMAO, μM | 3.8 (2.2, 8.0) | 4.8 (3.1, 7.9) | 0.30 |

CVD indicates cardiovascular disease; HDL-C, high-density lipoprotein cholesterol; LDL-C, low-density lipoprotein cholesterol; eGFR, estimated glomerular filtration rate; CVD, cardiovascular disease; ARB, angiotensin II receptor blockers; ACEIs, angiotensin-converting enzyme inhibitors; FMD, flow-mediated vasodilation; NID, nitroglycerine-induced vasodilation; TMAO, trimethylamine N-oxide. The results are presented as means and ± SD or medians with interquartile ranges.

**Table S5.** Clinical characteristics of the high TMAO group and the low TMAO group.

| Variables | Low TMAO  (n=167) | High TMAO  (n=166) | P value |
| --- | --- | --- | --- |
| Age, yr | 57±14 | 63±13 | <0.01 |
| Men (%) | 88 (52.7) | 108 (65.1) | 0.02 |
| Body mass index, kg/m^2^ | 25.4±4.4 | 25.2±3.9 | 0.71 |
| Heart rate, bpm | 73±13 | 70±11 | 0.04 |
| Systolic blood pressure, mmHg | 134±19 | 129±16 | 0.02 |
| Diastolic blood pressure, mmHg | 83±13 | 79±12 | 0.01 |
| Total cholesterol, mg/dl | 196±36 | 192±35 | 0.35 |
| Triglycerides, mg/dl | 141 (86, 209) | 116 (87, 177) | 0.13 |
| HDL-C, mg/dL | 58±16 | 59±17 | 0.81 |
| LDL-C, mg/dL | 116±28 | 108±30 | 0.02 |
| Creatinine, mg/dL | 0.76±0.17 | 0.88±0.42 | <0.01 |
| eGFR, mL/min/1.73m^2^ | 75.2±17.6 | 67.2±16.0 | <0.01 |
| Uric acid, mg/dL | 5.7±1.3 | 6.0±1.4 | 0.02 |
| Glucose, mg/dL | 110±26 | 108±21 | 0.46 |
| Hemoglobin A1c, % | 5.5±1.0 | 5.5±0.6 | 0.99 |
| Medical history, n (%) |  |  |  |
| Dyslipidemia | 117 (70.1) | 113 (68.1) | 0.69 |
| Diabetes mellites | 35 (21.0) | 40 (24.1) | 0.49 |
| CVD, n (%) | 19 (11.4) | 30 (18.1) | 0.08 |
| Current Smoking, n (%) | 36 (21.7) | 30 (18.1) | 0.41 |
| Medication, n (%) |  |  |  |
| Antihypertensive drugs, n (%) | 145 (86.8) | 155 (93.4) | 0.04 |
| Calcium channel blockers | 125 (74.9) | 123 (74.1) | 0.87 |
| ARB/ACEIs | 61 (36.5) | 89 (53.6) | <0.01 |
| α-Blockers, | 11 (6.6) | 10 (6.0) | 0.83 |
| β-Blockers | 7 (4.2) | 15 (9.0) | 0.07 |
| Diuretics, | 15 (9.0) | 26 (15.7) | 0.06 |
| Aldosterone antagonists | 32 (19.2) | 39 (23.5) | 0.33 |
| Lipid lowering drugs | 60 (35.9) | 66 (39.8) | 0.47 |
| Antidiabetic drugs, n (%) | 25 (15.0) | 25 (15.1) | 0.98 |
| Number of hypertensive drugs | 1.5±1.0 | 1.8±1.0 | <0.01 |
| FMD, % | 3.8±2.9 | 3.4±2.6 | 0.21 |
| NID, % | 12.1±5.6 | 11.3±6.1 | 0.23 |

TMAO indicates trimethylamine N-oxide; HDL-C, high-density lipoprotein cholesterol; LDL-C, low-density lipoprotein cholesterol; eGFR, estimated glomerular filtration rate; CVD, cardiovascular disease; ARB, angiotensin II receptor blockers; ACEIs, angiotensin-converting enzyme inhibitors; FMD, flow-mediated vasodilation and NID, nitroglycerine-induced vasodilation; The results are presented as means and ± SD or medians with interquartile ranges.

| Variables | Correlation coefficient (r) | P value | P for interaction |
| --- | --- | --- | --- |
| Male | -0.05 | 0.47 |  |
| Female | -0.15 | 0.09 | 0.24 |
| Age <65, year | -0.04 | 0.57 |  |
| Age ≧65, year | -0.15 | 0.65 | 0.95 |
| Dyslipidemia | -0.06 | 0.53 |  |
| Non-dyslipidemia | -0.13 | 0.05 | 0.62 |
| Diabetes | -0.05 | 0.66 |  |
| Non-diabetes | -0.12 | 0.06 | 0.57 |
| Current smoker | -0.11 | 0.38 |  |
| Non-current smoker | -0.11 | 0.07 | 0.95 |
| CVD | -0.01 | 0.98 |  |
| Non-CVD | -0.12 | 0.05 | 0.50 |

**Table S6.** Correlation between FMD and TMAO

FMD indicates flow-mediated vasodilation; TMAO, trimethylamine N-oxide; CVD, cardiovascular disease.

**Table S7.** Correlation between NID and TMAO

| Variables | Correlation coefficient (r) | P value | P for interaction |
| --- | --- | --- | --- |
| Male | -0.09 | 0.22 |  |
| Female | -0.11 | 0.19 | 0.64 |
| Age <65, year | -0.11 | 0.14 |  |
| Age ≧65, year | 0.03 | 0.70 | 0.49 |
| Dyslipidemia | -0.10 | 0.13 |  |
| Non-dyslipidemia | -0.13 | 0.19 | 0.69 |
| Diabetes | -0.19 | 0.11 |  |
| Non-diabetes | -0.08 | 0.22 | 0.45 |
| Current smoker | -0.24 | 0.06 |  |
| Non-current smoker | -0.08 | 0.20 | 0.39 |
| CVD | -0.05 | 0.74 |  |
| Non-CVD | -0.10 | 0.10 | 0.77 |

NID indicates flow-mediated vasodilation; TMAO, trimethylamine N-oxide; CVD, cardiovascular disease.
